# Rate of central corneal thickness changes in primary angle closure eyes: long-term follow-up results

**DOI:** 10.1186/s12886-021-01908-4

**Published:** 2021-03-22

**Authors:** Hae Min Park, Jiin Choi, Won June Lee, Ki Bang Uhm

**Affiliations:** 1 Department of Ophthalmology, Hanyang University College of Medicine, 222-1, Wangsimni-ro Seongdong-gu, Seoul, 04763 South Korea; 2 Department of Ophthalmology, Hanyang University Seoul Hospital, 222-1, Wangsimni-ro Seongdong-gu, Seoul, 04763 South Korea; 3 Office of Hospital Information, Seoul National University Hospital, Seoul, South Korea

**Keywords:** Glaucoma, Central corneal thickness, Corneal biomechanics, Primary angle-closure, Glaucoma management

## Abstract

**Background:**

Central corneal thickness (CCT) and its association with intraocular pressure, which is a pivotal parameter in glaucoma management, has previously been reported. In this study, we intended to investigate the long-term change of CCT in terms of rate in eyes with primary angle-closure (PAC). Additionally, we aimed to analyze events that could affect CCT.

**Methods:**

In this retrospective study, 26 patients with PAC who had a follow-up period of more than 5 years were analyzed. The rate of CCT changes from baseline was evaluated from the serial CCT measurements over the average follow-up period. The pattern of CCT change rate according to modes of treatment and history of angle-closure attack was analyzed using the repeated linear mixed model analysis.

**Results:**

A total of 52 eyes were enrolled. The CCT reduction rate of the entire study population was − 0.72 ± 0.22 μm/yr (*P* = 0.001) with statistical significance. The CCT thinning rate of the laser peripheral iridotomy (PI) group was − 0.53 ± 0.25 μm/yr (*P* = 0.034) and that of the surgical trabeculectomy group was − 1.32 ± 0.43 μm/yr (*P* = 0.002), and it was not statistically significant (*P* = 0.112). The rate of CCT thinning in patients with a history of acute angle-closure attack was − 0.81 ± 0.31 μm/yr (*P* = 0.009) and that in patients without an attack was − 0.63 ± 0.30 μm/yr (*P* = 0.001), and it was not statistically significant (*P* = 0.680). Baseline CCT appeared to be the only significant factor affecting the rate of CCT changes (*P* < 0.001).

**Conclusions:**

We found a significant reduction in CCT over a long observation period in PAC eyes. We also found that the rates of CCT reduction were not affected by different treatment modalities or acute angle-closure attacks. The analysis of long-term CCT changes in conjunction with baseline CCT would also be helpful in the clinical evaluation of the PAC patients.

**Supplementary Information:**

The online version contains supplementary material available at 10.1186/s12886-021-01908-4.

## Précis

Significant reduction in central corneal thickness (CCT) was observed over a long time period in primary angle closure eyes. The rates of CCT reduction were not affected by treatment modalities or angle closure attack but affected by baseline CCT.

## Background

As far as is known, intraocular pressure (IOP) is the only modifiable risk factor for glaucoma, which is a multivariate figure and a small error of any variable can result in under- or over-estimation, which may significantly affect treatment and prognosis [1, 2].

Primary angle-closure (PAC) is a more common form of glaucoma in Asians compared to other ethnicities. It is part of a broader disease spectrum that includes primary angle-closure suspect, PACS, primary angle-closure glaucoma, PACG, and PAC. PAC is known as a condition with physical narrowing of the anterior chamber owing to synechiae or appositional forces, which leads to progressive damage of the optic nerve. Of note, eyes with only physical narrowing of the anterior chamber and angles are known as PACS. PACS in the presence of raised IOP or peripheral anterior synechiae is termed PAC. It is known as PACG when the optic nerve or visual field is damaged. In the case of acute angle-closure, a rapid increase of IOP causes swelling and endothelial damage of the cornea, resulting in various biomechanical changes.

The importance of CCT in glaucoma is well established. It is widely accepted that true IOP with the thinner cornea is higher than its measurement, and vice versa. Famously, the ocular hypertension treatment study, European glaucoma prevention study, and Barbados eye study showed that CCT could be a risk factor for the development and progression of glaucoma disease [3–6]. Furthermore, multiple reports, including Early Manifest Glaucoma Trial and Collaborative Normal-Tension Glaucoma Study Group, have analyzed that IOP alone cannot slow the progression of glaucomatous damage [7–9]. Thereby, the concept of monitoring of IOP along with other ocular parameters has emerged.

Corneal biomechanical properties were one of the important ones to be discussed. This is mainly because it has a major effect on the accurate measurement of IOP. In the past, glaucoma studies regarding corneal characteristics, mainly CCT, were conducted mostly in open-angle, normal-tension glaucoma, or a heterogeneous cohort. Although there are many reports about CCT that focus on PAC, recent attention has been centered on corneal hysteresis (CH). It has been reported that CH is related to CCT and some up-to-date literature reports its association with IOP, glaucomatous changes, long-term glaucoma medications, and so on. In other words, corneal biomechanics, including CH, can be an alternative for the evaluation of overall ocular biomechanics. This presents as a possible indicator of glaucomatous damage [10–21]. However, in most clinics, the instrument that measures CH is not readily available and yet to be clinically used for glaucoma evaluation.

CCT is important in the clinical evaluation of glaucoma. We believe that a proper understanding of CCT changes in PAC eyes with time is crucial since PAC itself and even its treatments can induce long-term changes in the cornea. Previously, many researchers have studied the association between CCT and glaucoma [3–9]. However, most of the studies were conducted in non-Korean populations and were short-term and cross-sectional studies. Although some longitudinal studies were also carried out, many were short-term studies with no significant results or that had sparsely collected data. Moreover, most reports were heterogeneous cohorts and included various types of glaucoma [22].

This study aimed to investigate the long-term rate of CCT change in eyes with PAC. Longitudinal, long-term observation of CCT and the concept of analyzing it by rate is not unprecedented. Nonetheless, the fact that our data are unique to the PAC eyes of the Korean population and analyzes data that was consistently cumulated every year makes a difference that only this study can present.

## Methods

The Institutional Review Board of Hanyang University Hospital approved this study (IRB No. 2020–04–057-001). The study design followed the tenets of the Declaration of Helsinki for biomedical research [23].

### Subjects

A total of 27 patients within the PAC disease spectrum who were followed-up for more than five years at the Department of Ophthalmology of Hanyang University Hospital from January 2002 to December 2010 were enrolled in this study and their medical charts were retrospectively reviewed. Patients with PAC disease spectrum, either unilateral or bilateral, were included in the study. PAC diagnosis was based on gonioscopic findings, where posterior trabecular meshwork was not visible on non-indentation gonioscopy for at least 2 quadrants at the primary position. Clinical definitions of PAC and its disease spectrums are further described in the oncoming section. Referral status, history of angle-closure attack, certain glaucoma medications were not part of either inclusion or exclusion criteria. Exclusion criteria included secondary angle-closure due to many possible causes, such as neovascular, uveitic glaucoma or trauma, corneal disorders that prevent accurate measurements, trauma, and other ocular disorders after intraocular surgery. Patients with less than a 5-year follow-up period and those with a previous history of ocular surgeries other than glaucoma-related surgeries were also excluded from the study. Patients with a history of cataract surgery were not excluded.

All subjects underwent a complete ophthalmologic examination, including visual acuity testing, manifest refraction assessment, slit-lamp examination, IOP measurements using Goldmann applanation tonometry, gonioscopy, dilated fundus examination, axial length measurement (IOLMaster; Carl Zeiss Meditec, Dublin, Ca, USA, Aviso; Quantel medical, Quebec, Canada), stereo-disc photography and red-free RNFL photography (TRC-50X; Topcon Corporation, Tokyo, Japan, F-10; Nidek, Gamagori, Japan), and Swedish interactive thresholding algorithm (SITA) 30–2 perimetry (Humphrey Field Analyzer II; Carl Zeiss Meditec, Jena, Germany). Visual acuity was measured with a standardized Korean eye chart (standard chart distance at 3 m), and measurements were recorded in a decimal system. Manifest refraction was measured with an auto refractometer and was never manually measured. Gonioscopy and dilated fundus exam was performed by a single glaucoma specialist (KBU). Volk 4 mirror gonio lens and Volk superfield lens were used for gonioscopy and dilated fundus examinations. The CCT measurements were taken using an ultrasound pachymeter (Tomey SP-3000; Tomey Corporation, Nagoya, Japan) by the same technician, recording a mean of ten consecutive readings. The above-explained ophthalmologic examinations were performed every year for all study patients.

### PAC and its disease spectrum

The latest classification scheme by the International Society of Geographical and Epidemiological Ophthalmology (ISGEO) describes features of the PAC spectrum (PACS, PAC, PACG). Accordingly, PACS was defined as present in an eye with an “occludable angle” with normal IOP, less than 21 mmHg. The occludable angle was defined as when the posterior trabecular meshwork was not visible on the non-indentation gonioscopy for at least two quadrants at the primary position. PAC was defined as PACS eye with increased IOP, trabecular obstruction, such as peripheral anterior synechiae (PAS), increased IOP, iris whirling, and glaucomflecken. Both PAC and PACS should not have had glaucomatous optic damage. PACG was defined as the presence of glaucomatous optic neuropathy with compatible visual field loss in an association with occludable angle [24].

In this study, a single glaucoma specialist (KBU) made clinical diagnoses using a non-indentation gonioscopy. All surgical and laser treatment decisions were made and undertaken by a glaucoma specialist (KBU). The laser peripheral iridotomy (PI) was done for patients with acute angle-closure by the trained doctor mentioned above. Furthermore, prophylactic PI was performed for patients without an attack, who were considered to have PAC in a broad sense. Trabeculectomy was performed in cases with severe corneal edema or failed PI, such as persistently high IOP or progression of glaucomatous damage after PI. After PI and/or trabeculectomy IOP of all included patients was controlled within the normal range. Baseline studies were performed after the corneal status was normalized, following laser peripheral iridotomy and/or trabeculectomy.

PI was performed in a standard manner. After administration of 2% pilocarpine eye drops, PI was done using argon and Nd:YAG lasers, sequentially. The argon laser was used to irradiate the iris, using an Abraham lens. First, 3–6 pulses at a power of 200 mW and a spot diameter of 200 μm with a duration of 0.2 s for iris extension, was performed. Then, 10–40 pulses at 800-mW power, the spot diameter of 50 μm, and a duration of 0.05 to create perforation in the iris were done. Irradiations were applied to the superior iris in order to avoid corneal complications. Finally, pulses of 3.5 to 5.0 mJ of the Nd:YAG laser were used for complete perforation of the wound. Preoperatively, the administration of anti-glaucoma medications and steroid medications were used for one week. When IOP was not adequately controlled, even after surgical or laser procedures, additional anti-glaucoma medications were prescribed by the single glaucoma specialist (KBU).

### Calculation of central corneal thickness changing rates & statistical analyses

All statistical tests were performed using SAS version 9.4 (SAS Institute Inc., Cary, NC, USA). Patient characteristics with continuous variables were expressed as the mean ± SD, and nominal variables were expressed as frequencies and percentages. The normality of the distribution of the CCT scores was verified using the Shapiro-Wilk test. The rate of CCT changes from baseline was determined from the serial measurements using repeated linear mixed model analysis (expressed in μm per year), with a restricted maximum likelihood estimation. Fixed effects were treatment group (trabeculectomy vs. PI and angle-closure attack vs. no attack), time of measurement, and the treatment group by time interaction. The rates of change were compared among groups through testing of the interaction term in the linear mixed models. In this model, the treatment by time interaction was not statistically significant. It indicates that there were no differential changes in CCT over time, depending on treatment groups. The covariance pattern between the repeated measurements was assumed to be compound symmetry. We considered different forms of the terms of the random effects ranging from the simplest model with no random effects to the largest model with random intercepts and random slopes. We computed the Akaike information criterion (AIC) for a set of candidate models with different forms of random effects and selected the one with the smallest AIC value indicating a better fitting model. Finally, we applied an eye-specific random-effects model. Additionally, we analyzed the associated CCT change rate in PAC patients with clinicopathological factors of interest using a linear mixed model, and estimate, SE, and its *P*-values were calculated. The level of significance was set at P-value < 0.05.

## Results

The study evaluated 54 eyes of 27 patients with PAC. Two eyes of one patient, which developed bullous keratopathy due to failure of IOP control, were excluded. Finally, 52 eyes of 26 patients were analyzed.

### Clinical demographics

Table 1 shows the clinical demographics of all patients at the time of enrollment. A total of 40 eyes underwent PI only. A total of 12 patients had trabeculectomy. The latter group included patients who underwent a PI before trabeculectomy. Twenty-three eyes had a history of angle-closure attack.

**Table 1:** Participant demographics

	*N* = 26, 52 eyes
Age	63.4 ± 8.7
Sex (M:F)	2:24 (7.7%:92.3%)
Initial IOP	29.6 ± 18.2
MD	−6.47 ± 7.24
Axial length	22.55 ± 0.73
Baseline CCT	549.1 ± 29.2
Iridectomy only / Trabeculectomy	40/12 (76.9%/23.1%)
Angle closure attack / no attack	23/29 (44.2%/55.8%)
Follow up duration (months)	94.5 ± 28.7
Examination number	7.0 ± 2.1
Patients with diabetes (%)	17/26 (65%)
Patients with hypertension (%)	5/26 (19%)
History of smoking	1/26 (3.8%)

*IOP* indicates intraocular pressure, *MD* mean deviation from automated visual field exam, *CCT* central corneal thickness

The average number of the examinations was 7.0 ± 2.1 (range, 4–10) over a mean follow-up period of 94.5 ± 28.7 months (range, 66–134 months). Most of the study population consisted of females (92.3% of all patients) with a mean age of 63.4 ± 8.7 years (range, 48–78 years). The baseline CCT was 549.1 ± 29.2 μm (range, 484–619 μm), and mean presenting IOP was 29.6 ± 18.2 mmHg (range, 6–64 mmHg).

After PI and/or trabeculectomy, all of the included patients maintained stable IOP status without additional surgical or laser treatments to reduce intraocular pressure. Only 13 eyes required more than 1 class of anti-glaucoma medication, whereas 24 eyes needed none. Among the 13 eyes, 5 eyes were treated with all 3 types of IOP-lowering medications, and 8 eyes were treated with 2 types. In detail, 4 eyes were treated with a combination of beta-blocker and prostaglandin analogs, 3 eyes with beta-blocker and carbonic anhydrase inhibitor, and 1 eye with alpha-2-agonist and beta-blocker. Others were under topical anti-glaucoma monotherapy during the follow-up period. Monotherapy with alpha-2- agonist in 3 eyes, Beta-blocker with 8 eyes, and prostaglandin analogs in 5 eyes. In this study, about 10 patients underwent phacoemulsification and intraocular implantation surgeries after baseline review. Cataract surgery was performed to improve visual acuity and not to reduce IOP.

### Central corneal thickness change rate

In each group, there was a statistically significant reduction in CCT, as indicated in Supplementary Table 1. The CCT thinning rate of all enrolled patients was − 0.72 ± 0.22 μm/yr, and it was a statistically significant reduction (*P* = 0.001).

**Fig. 1:**
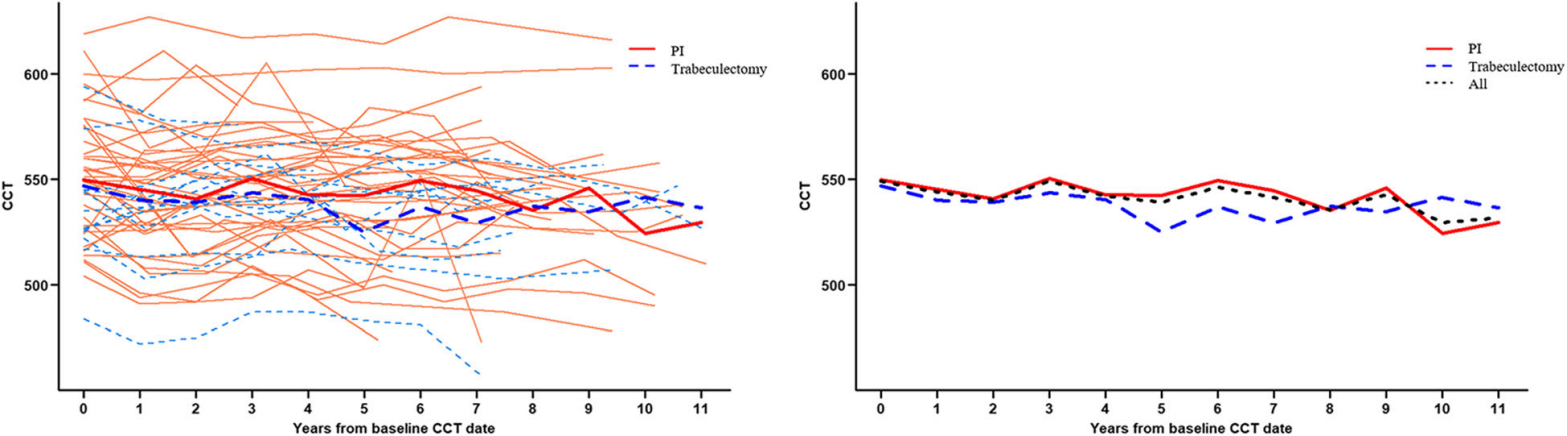
Comparison of central corneal thickness changes between the trabeculectomy and laser peripheral iridectomy only groups over time. Changes of central corneal thickness (CCT) over the 11 years. The CCT of the trabeculectomy group is lower than that of the laser peripheral iridectomy (PI) only group, which was revealed to be statistically insignificant (*P* = 0.112). Note: bold lines represent the mean value

**Fig. 2:**
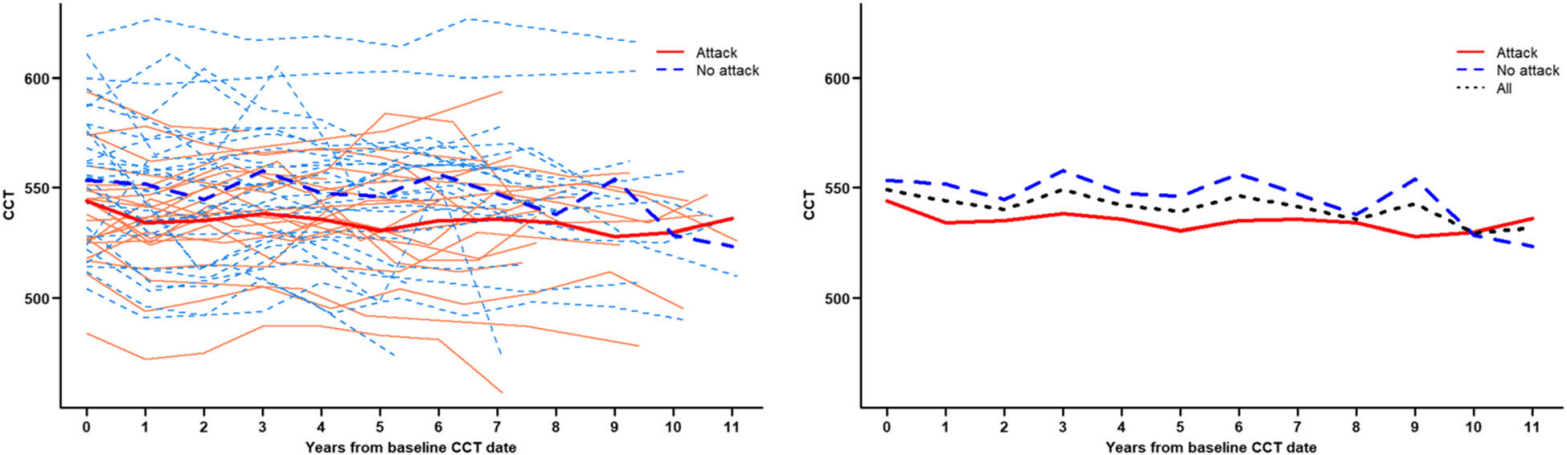
Comparison of central corneal thickness changes between the two groups: with angle closure attack and without an attack. Changes of central corneal thickness (CCT) over 11-year period. The CCT of the study group with an acute angle closure attack seems to be lower than that of the study group without an attack. Statistical analysis showed there was no statistical significance between the central corneal thickness thinning rate of the two groups (*P* = 0.680). Note: bold lines represent the mean value

Overall, the CCT thinning rate in each of the two groups was statistically significant. The CCT thinning rate of the PI group was − 0.53 ± 0.25 μm/yr, that of the trabeculectomy group was − 1.32 ± 0.43 μm/yr (*P* = 0.034 and *P* = 0.002, respectively). However, the statistical analysis showed that a higher CCT thinning rate of the trabeculectomy group was not statistically significant (*P* = 0.112) (Table 2 and Fig. 1). Similar results were observed in CCT comparison with regard to the presence of an angle-closure attack. The rate of CCT thinning in patients with a history of acute angle-closure attack was − 0.81 ± 0.31 μm/yr and that in patients without an attack was − 0.63 ± 0.30 μm/yr (*P* = 0.009 and *P* = 0.001, respectively). There was no statistically significant difference in the CCT thinning rate between the two groups (*P* = 0.680). (Table 2 and Fig. 2).

**Table 2:** Central corneal thickness change rate according the type of operation and to the presence of acute angle closure attack

	**According to the type of operation**			
	**Total**	**PI only**	**Trabeculectomy**	***P*-value**^**a**^
CCT thinning rate	−0.72 ± 0.22 (*P* = 0.001)	−0.53 ± 0.25 (*P* = 0.034)	−1.32 ± 0.43 (*P* = 0.002)	0.112
	**According to the presence of angle closure attack**			
	**Total**	**Attack (+)**	**Attack (−)**	***P*-value**^**b**^
CCT thinning rate	−0.72 ± 0.22 (*P* = 0.001)	−0.81 ± 0.31 (*P* = 0.009)	−0.63 ± 0.30 (*P* = 0.001)	0.680

*CCT* indicates central corneal thickness, *PI* laser peripheral iridotomy

^a^ Repeated Measures Linear Mixed Model: Duration as continuous variable & Operation

^b^Repeated Measures Linear Mixed Model: Duration as continuous variable & angle closure attack

Figures 1 and 2 show CCT changes during the follow-up period, with the maximum being close to 11 years. It demonstrates the changes of CCT over time in all study patients, and that is divided by subgroups (trabeculectomy vs. PI, and angle-closure attack vs. no attack). The mean CCT changes were schematically drawn to compare the total study groups and the subgroups easily.

The results of the analysis for identifying factors associated with the CCT change rates are summarized in Table 3. Only baseline CCT was associated with the rates of its thinning (*P* < 0.0001). In other words, a higher baseline value was associated with a higher rate of CCT reduction. However, in this statistical analysis, CCT change rate was not significantly associated with age (*P* = 0.297), sex (*P* = 0.231), IOP (*P* = 0.295), type of intervention (*P* = 0.913), and presence of angle-closure attack (*P* = 0.238).

**Table 3:** Factors associated with central corneal thickness change rate in primary angle closure patients

	Estimate	SE	CI	P-value^a^
Age	−0.27	0.25	(−0.77, 0.24)	0.297
Sex: Female (vs. Male)	−11.21	9.23	(−29.81, 7.40)	0.231
IOP	−0.27	0.26	(−0.77, 0.24)	0.295
MD	−0.08	0.26	(−0.59, 0.43)	0.768
Axial length	5.25	2.99	(−0.77, 11.28)	0.086
Angle closure attack (vs. No attack)	−5.32	4.45	(−14.28, 3.64)	0.238
Type of intervention : Trabeculectomy (reference: PI)	−0.59	5.38	(−11.40, 10.22)	0.913
Baseline CCT	0.81	0.07	(0.67, 0.94)	**< 0.001**
Duration	−0.45	0.34	(−1.13, 0.23)	0.195

*IOP* intraocular pressure, *MD* mean deviation from automated visual field exam, *PI* laser peripheral iridotomy, *CCT* central corneal thickness

^a^Repeated Measures Linear Mixed Model

## Discussion

Biometrics of PAC eyes has been a subject of study for many years. Recent interest in corneal biomechanics is due to its influence on the accuracy of IOP measurements [25]. In this retrospective study, we found a marked reduction of CCT in all PAC patients enrolled in this study. However, no significant difference was observed regardless of the acute angle-closure attack and modes of glaucoma treatment. Previous longitudinal CCT studies show similar findings, but with sparse data collection [22, 26–29]. Most analyses were done with data collected only at two time points in which initial data were collected at baseline and the other collected at the end of the follow-up period. Instead, this study analyzed data that were collected every year. Although the results did not show any remarkable differences from previous results, earnest collection of data for the long-term can better describe the trend of changes. We believe that this study has its originality in that it conducted a long-term evaluation of CCT changes in PAC patients with a meticulous yearly collection of data.

The study by Aghaian et al. and many other studies reported CCT variability among different ethnicities. Thus, many CCT studies with various ethnicities were published [28, 30, 31]. Nonetheless, studies specific to the Korean population are particularly lacking, especially in the context of PAC eyes [32–34]. We only managed to find one study that reports a cross-sectional comparison of CCT in PACG, POAG, NTG, and the normal population [32]. As far as we understand, this is the first longitudinal study of CCT changes in PAC eyes of Korean patients.

Several studies in the past, mostly cross-sectional, have compared CCT in normal controls and other glaucoma subtypes, including PAC. The results of the studies mostly suggested that there was no statistically significant difference in CCT among glaucoma subtypes and normal controls [35–37]. A large population study in Beijing conducted by Xu et al. also suggested no significant difference between glaucomatous and normal eyes [38]. Furthermore, it was also reported that CCT of PACG eyes was similar to that of NTG or normal eyes in Korean subjects [26].

Comparable to our study designs, some studies have studied CCT changes in PAC eyes only. Chen et al. studied corneal status in the PAC disease spectrum. In this study, PAC eyes with a history of ACG attack were compared with fellow PAC eyes without attack and found no significant difference in CCT between the two groups [39]. Although previous studies published statistically significant results with large study groups, the weakness was that they were short-term. However, in real clinics, glaucoma patients require long-term management of IOP, and it is a common understanding that it should be analyzed with CCT. Therefore, we believe being conscious of long-term corneal changes would be more applicable in the clinical assessment of glaucoma. The corneal characteristics are changeable with aging and corneal insults, such as surgeries, angle-closure attacks, and topical medications.

In this study, the final mean CCT and its change rate were not significantly different between the PI and trabeculectomy groups. The same result was observed for eyes with and without acute angle-closure attack. This is consistent with few previous studies. Pillunat et al. investigated corneal biomechanical changes after trabeculectomy and showed that despite a decline in IOP, CCT was not altered [40]. A few other studies suggested similar findings with PI [41, 42].

Instead of CCT, more up-to-date reports focus on CH and its association with glaucoma [10–21]. CH is a dynamic property that reflects the deformability of the cornea, which represents its capacity to endure IOP fluctuations. CH reflects biomechanical properties of the corneal extracellular matrix, in which imbalanced remodeling and degradation induce a fibrotic response that ultimately leads to tissue stiffening. There are several reports about CH and CCT relationship [10, 13, 16, 18]. It was widely reported that CCT thinning after aging or use of long-term topical anti-glaucoma medications, especially prostaglandin analogs, may be related to degradation of the corneal stromal extracellular matrix [43, 44]. More importantly, a moderate positive correlation between the two parameters, and a negative correlation between CH and IOP has also been reported [12, 45]. This suggests that CCT reduction in this study may be related to extracellular matrix changes, although we were unable to determine the relevant factors or possible causes from this study. However, there are limitations to this interpretation.

The relationship between CCT and CH is very complex that more needs to be clarified, and many published reports showed a non-consistent relationship between the two. However, a possible effect of CH on IOP has been consistently published. Some publications showed that considering both CH and CCT is better for accurate IOP evaluation than using CCT alone [12, 16, 46]. Further studies focusing on this corneal parameter may be more intriguing and helpful in further understanding PAC eyes.

Multiple studies have shown irreversible corneal endothelial damage caused by surgeries, laser procedures, and even topical medications [40–42, 47–50]. In relevance to the field of glaucoma, many researchers have studied associations between corneal endothelial loss with glaucoma subtypes. Shiota et al. investigated corneal endothelial status across subtypes of angle-closure glaucoma. They found that the previous acute angle closure and chronic PACG had significantly lower endothelial cell density (ECD) [51]. At the same time, there are conflicting studies. Varadaraj et al. and Verma et al. studied endothelial changes in the PAC disease spectrum, including eyes with a history of acute angle closure attack and found no significant difference in all groups [52, 53]. Interestingly, a recent study of a rat model showed eyes with acute angle closure had lower ECD at first, but showed a gradual resolution once IOP stabilized to normal levels [54]. It may be more useful to assess endothelial changes since CCT does not reflect the overall corneal status. Unfortunately, we were not able to perform endothelial studies on our study population owing to the retrospective nature of this study. A long-term evaluation of CCT in conjunction with ECD changes is required for future studies, and assessment of corneal status with various parameters will enrich future study results.

The repeated mixed model in this study showed that baseline CCT was the only factor associated with the rate of its reduction. Two possible mechanisms can explain such a finding. First, eyes with thick cornea can be intrinsically prone to CCT thinning. This mechanism requires further study or statistical analysis that directly compares the CCT thinning rate between eyes with a thin cornea and thick cornea. Second, corneal edema caused by an acute angle-closure attack or anti-glaucoma procedures can be another possible explanation. Corneal edema can quickly resolve as IOP normalizes, which in turn, can overestimate CCT thinning rate. Correlation with the interval between the acute insult and CCT measurement will be helpful to clarify this finding further.

The primary strength of this study is that the longitudinal change of CCT in the extended time was evaluated, with the maximum follow-up duration being close to 11 years. Another strength is that we evaluated CCT with account for events that can affect the corneal status, such as acute angle-closure attack and surgeries. We are aware of the numerous publications with notable results that studied CCT changes in normal and different glaucoma types [48–50]. However, these studies had a small sample size and, more importantly, had short observation periods, ranging from months to 4–5 years. The cornea is a structure that is prone to change over time. It can change with aging, long-term topical medication use, surgeries, and even diurnally [26, 43, 55, 56]. Considering this, we believe that a long-term investigation of CCT concerning acute events is more applicable in glaucoma evaluation than cross-sectional studies of CCT.

Despite the distinct merits of this study entails, there are some limitations. One is that the study has no data regarding CCT changes with age and that in the normal controls or other forms of glaucoma. Many published reports are showed a decrementing trend of CCT with age, but some show contradictory results. Pang et al. and Day et al. studied CCT and its relationship with glaucoma in Asian and East Asian patients; their results showed statistically significant CCT reduction with increasing age. In this study, the CCT change rate and age were found to have no association. However, since the study only included PAC eyes, comparison with normal controls or other forms of glaucoma is required for a more fair comparison. Another limitation is that other possible confounding factors were not considered in this study. It has been previously reported that systemic diseases, such as diabetes and some anti-glaucoma medications, can affect corneal biometrics. Particularly, prostaglandin analogs, such as latanoprost and travoprost, [48–50, 57] were associated with thinning of CCT in both short-and long-term studies. However, owing to the extensive study period, information on the duration of usage, changes in regimen was difficult to follow-up, as well as to statistically analyze. For this reason, these possible contributing factors were ignored in this study and further study will be required to assess its conduciveness [58]. Additionally, larger sample size will be required for further studies. This may have been because the study was retrospectively designed and included patients with a very long-term follow-up period, which makes it difficult to collect a sufficient sample size. Lastly, as aforementioned, other corneal parameters, such as ECD, were not taken into consideration owing to the retrospective nature of this study. Evaluation of PAC eyes with various parameters would be more interesting for future study and more valuable in understanding the true changes in corneal biomechanics of PAC eyes.

## Conclusions

To conclude, we found a significant decline in CCT over a long time-period in PAC eyes. Baseline CCT appeared to be the only significant factor affecting the rate of changes, not by treatment modalities or history of ACG attack. Analysis of CCT changes by its baseline, and in conjunction with other corneal parameters are needed for a better understanding of PAC eyes.

## Supplementary Information

**Additional file 1.**

## Data Availability

The datasets used and/or analysed during the current study available from the corresponding author on reasonable request.

## Abbreviations

CCT: Central corneal thickness
PAC: Primary angle closure
PI: Laser peripheral iridotomy
IOP: Intraocular pressure
PACS: Primary angle closure suspect
PACG: Primary angle closure glaucoma
ISGEO: International Society of Geographical and Epidemiological Ophthalmology
CH: Corneal hysteresis
