# Supplementary material for: Rate of central corneal thickness changes in primary angle closure eyes: long-term follow-up results
Source: BMC Ophthalmol. 2021 Mar 22;21:145. doi: 10.1186/s12886-021-01908-4 (PMC7986557; doi:10.1186/s12886-021-01908-4)
Supplement: Supplementary file 1 — Additional file 1. [file 12886_2021_1908_MOESM1_ESM.pdf]

Rate of Central Corneal Thickness Changes in Primary Angle Closure Eyes: Long-term Follow-up Results

Hae Min Park, MD[1,2] , Jiin Choi, PhD[3],Won June Lee, MD, PhD[1,2*], Ki Bang Uhm, MD, PhD[1]

[1]Department of Ophthalmology, Hanyang University College of Medicine, Seoul, Korea

[2]Department of Ophthalmology, Hanyang University Seoul Hospital, Seoul, Korea

[3]Office of Hospital Information, Seoul National University Hospital, Seoul, Korea

***Correspondence: wonjunelee@hanyang.ac.kr**

Correspondence to: Won June Lee, MD, PhD

Department of Ophthalmology, Hanyang University College of Medicine

Department of Ophthalmology, Hanyang University Seoul Hospital,

222-1, Wangsimni-ro Seongdong-gu, Seoul Korea (04763)

Tel: +82-2-2290-8570, Fax: +82-2-2291-8517

E-mail: **wonjunelee@hanyang.ac.kr**

Supplementary table 1. Central corneal thickness (μm) at each patient visit

Data	Time	Total	Operation		Attack	
			PI	TLE	Attack (+)	Attack (-)
		(p = 0.001)	(p = 0.030)	(p = 0.004)	(p = 0.006)	(p = 0.061)
CCT	1st (n=52)	549.1±4.1	549.8±4.6	546.9±8.6	543.9±5.4	553.2±5.7
	1year (n=44)	543.4±4.1	544.6±4.7	539.7±8.7	535.1±5.5	550.0±5.8
	2year (n=40)	544.5±4.2	545.9±4.7	539.5±8.8	537.6±5.6	550.0±5.8
	3year (n=38)	547.2±4.2	549.1±4.7	540.8±8.9	539.3±5.6	553.5±5.9
	4year (n=32)	546.8±4.3	547.3±4.9	544.1±8.8	540.9±5.7	551.3±6.0
	5year (n=36)	543.6±4.2	545.2±4.7	538.1±9.2	536.5±5.7	549.3±5.9
	6year (n=32)	544.9±4.3	548.5±4.9	534.4±8.8	535.6±5.7	552.8±6.0
	7year (n=30)	543.0±4.3	545.8±4.8	532.8±9.2	535.8±5.8	548.9±6.0
	8year (n=22)	542.9±4.5	545.1±5.1	535.6±9.4	535.0±5.9	549.6±6.5
	9year (n=14)	537.0±4.9	540.7±5.6	526.2±9.8	529.2±6.8	543.1±6.8
	10year (n=10)	538.7±5.3	539.9±6.2	534.4±10.3	531.5±6.8	544.4±8.1
	11year (n=6)	536.6±6.2	537.1±7.4	533.4±11.4	533.9±7.6	533.3±10.4

CCT, central corneal thickness ; PI, laser peripheral iridotomy; TLE trabeculectomy.

Scores are presented by least squared means±se.
